# Information Overload in Emergency Medicine Physicians: A Multisite Case Study Exploring the Causes, Impact, and Solutions in Four North England National Health Service Trusts

**DOI:** 10.2196/19126

**Published:** 2020-07-27

**Authors:** Laura Sbaffi, James Walton, John Blenkinsopp, Graham Walton

**Affiliations:** 1 Information School University of Sheffield Sheffield United Kingdom; 2 Leeds Teaching Hospitals Trust Leeds United Kingdom; 3 North Tees and Hartlepool NHS Foundation Trust Stockton-on-Tees United Kingdom; 4 Centre for Information Management Loughborough University Loughborough United Kingdom

**Keywords:** emergency medicine, information overload, physicians, national health care system

## Abstract

**Background:**

Information overload is affecting modern society now more than ever because of the wide and increasing distribution of digital technologies. Social media, emails, and online communications among others infuse a sense of urgency as information must be read, produced, and exchanged almost instantaneously. Emergency medicine is a medical specialty that is particularly affected by information overload with consequences on patient care that are difficult to quantify and address. Understanding the current causes of medical information overload, their impact on patient care, and strategies to handle the inflow of constant information is crucial to alleviating stress and anxiety that is already crippling the profession.

**Objective:**

This study aims to identify and evaluate the main causes and sources of medical information overload, as experienced by emergency medicine physicians in selected National Health Service (NHS) trusts in the United Kingdom.

**Methods:**

This study used a quantitative, survey-based data collection approach including close- and open-ended questions. A web-based survey was distributed to emergency physicians to assess the impact of medical information overload on their jobs. In total, 101 valid responses were collected from 4 NHS trusts in north England. Descriptive statistics, principal component analysis, independent sample two-tailed *t* tests, and one-way between-group analysis of variance with post hoc tests were performed on the data. Open-ended questions were analyzed using thematic analysis to identify key topics.

**Results:**

The vast majority of respondents agreed that information overload is a serious issue in emergency medicine, and it increases with time. The always available culture (mean 5.40, SD 1.56), email handling (mean 4.86, SD 1.80), and multidisciplinary communications (mean 4.51, SD 1.61) are the 3 main reasons leading to information overload. Due to this, emergency physicians experience guideline fatigue, stress and tension, longer working hours, and impaired decision making, among other issues. Aspects of information overload are also reported to have different impacts on physicians depending on demographic factors such as age, years spent in emergency medicine, and level of employment.

**Conclusions:**

There is a serious concern regarding information overload in emergency medicine. Participants identified a considerable number of daily causes affecting their job, particularly the traditional culture of emergency departments being always available on the ward, exacerbated by email and other forms of communication necessary to maintain optimal, evidence-based practice standards. However, not all information is unwelcome, as physicians also need to stay updated with the latest guidelines on conditions and treatment, and communicate with larger medical teams to provide quality care.

## Introduction

### Background

The concept of information overload has existed long before the term was first defined in the 20th century. The writer of Ecclesiastes 12:12 in the third century before Christ was complaining that “of making books there is no end.” In the first century, Anno Domini, the Roman philosopher Seneca noted how “the abundance of books is distraction” [1]. Hence, information overload has been experienced by people long before the appearance of today's digital gadgets and social media in the work environment and at home. Most people appreciate the freedom that comes with choice and the many channels of media that saturate the information world. However, a 2016 Pew Research Center survey [2] showed that around 20% of the American population experienced overload and unease in looking for information. According to this study, those with more access to the internet are more likely to express worries about information overload and report difficulty in finding the information they need. Moreover, many institutions such as government agencies, schools, banks, and health care services require a lot of data and information gathering from their customers to process their requests, which contributes to stress and anxiety [2]. In the United Kingdom, a recent survey conducted by TeleWare on 2000 employees revealed that 36% of employees admitted that they had wasted a lot of their working day attempting to resolve an issue where they have forgotten valuable information. A similar number (34%) explained that forgetting information has led them to deal ineffectively with customers, suppliers, or clients. Around a quarter have missed important deadlines (26%) or let their colleagues down (25%) because they did not have the necessary information in the front of their mind [3].

The idea of having every piece of information at hand in any given situation is undoubtedly tempting and, in critical working situations, mandatory [4]. However, this is becoming increasingly unrealistic because of the large volume of data and information. An understanding of the current situation may be gathered by setting information overload in the theoretical framework provided by Floridi [5], whose view entails the definition of 3 main periods in the advancement of humanity: *prehistory*, before recorded information; *history*, when society was aided by recorded information; and *hyperhistory*, where society is shaped and dependent on information and communication technologies. The spreading of the *onlife* human condition, whereby lives are lived both online and offline in an *infosphere*, mirrors the progression of society through hyperhistory. This drastic shift in how people perceive their existence has raised a number of issues, including information overload in a more contemporary, Information and Communication Technology (ICT)-centered lens.

This study aimed to explore the impact of information overload on emergency medicine physicians (EMPs) working across 4 National Health Service (NHS) trusts in north England. The focus was to explore physicians’ views on the impact of information overload, including the extent of the impact, whether it had increased, and whether any preventative measures were taken to address information overload. The cross-disciplinary research team included an EMP, 2 academics, and an NHS clinical effectiveness adviser who oversaw the study.

### Literature Review

#### Information Overload in the Workplace

The concept of information overload became generally known in the early 1970s, after the publication of Toffler’s book on society’s deep organizational changes destined to cause shock and disorientation in people [6]. This initial idea of information overload refers to the difficulty a person can have understanding an issue and making errors caused by the presence of too much information. A later interpretation of the concept, in the 1990s, saw information overload defined as “occurring when the information processing demands on an individual's time to perform interactions and internal calculations exceed the supply or capacity of time available for such processing” [7].

Recent research has demonstrated how there cannot be a unifying definition of the concept, as the context in which it is used changes all the time [8,9]. There is however an agreement that information overload occurs when the information received becomes a burden rather than a help for users [10]. A survey conducted in 2016 by the Chartered Institute of Management Accountants showed how information overload was the main contributor to poor decision making in business globally [11]. Together with weakened decision making, information overload can also lead people to feel unnecessarily overwhelmed and stressed. Recent research has shown that people presenting with large amounts of information while performing search tasks tend to experience a sense of being overwhelmed and even display an increased heart rate, which leads to increased stress levels [12]. In the workplace environment, information overload can lead to a lack of engagement and loss of productivity [13-15]. The increase in mobile technologies has exacerbated this issue. The overload effects associated with the use of mobile information technologies in the workplace have become increasingly dominant and can translate into a considerable reduction in job satisfaction [16,17]. This is mostly because of the perception of immediacy that people have in connection with such devices. It is assumed that emails, text messages, and notifications should be read and answered quickly [18]. Several studies have demonstrated that training in an email or a social media protocol can be effective as a means to reduce information overload [9,19]. Interestingly, people of all ages and backgrounds are affected by this problem in every aspect of their working (and personal) life [9]. However, the same authors stated how the literature tends to focus on the workplace as a whole rather than on job specifications and particular skills required in certain occupations. The same view is shared by Kalman [20], who stated that “the workplace pressures attributed to information overload are very real, the concept itself is vague and poorly deﬁned.”. It is however worth noting that recent research has shown how information overload has the potential to enhance people’s cognitive abilities and help them process more information in less time, particularly if the right visualization tools (eg, charts and tables instead of text) are applied in the correct contexts [21]. There are benefits in assimilating larger amounts of information, and the human mind can adapt in time to the increased influx, but it is also bound to manifest side effects such as psychological disturbances and neurological problems along the way [22].

#### Information Overload and Health Care Professionals

When health care professionals experience information overload, at least one extra layer of concern must be taken into account: the consequence on patient care. Several studies have looked at the effects of information overload on nurses [23,24]; however, few have focused on information overload as experienced in general by health care professionals [25-27] and even less have approached the impact on patient care. Laker et al [28] argued how it could prevent practitioners from seeing the most important details while examining patients. Nevertheless, it is not the *right* information that is overwhelming, as that, if readily available at the point of care, would lead to effective patient care. It is the *background noise* information and other confounding factors (eg, multiple sources of information and conflicting or irrelevant information) that cause disruptions [29].

Klerings et al [27] agreed with this view and suggested solutions to overcome information overload and manage both health care information and patient care effectively. They mentioned different solutions based on the main overload trigger mechanisms, such as “technological solutions; creation or adaption of specific content types; improving health literacy; and creating or strengthening the roles of human intermediaries.”

Most likely, the situation is destined to get worse in this era of information expansion. As Yamamoto explained in 2016 [30], health care professionals are already overloaded by the information currently available, but the circumstances will worsen because of 3 key aspects: (1) the need to stay up to date from a technological and knowledge-based point of view; (2) the diversification of medical needs resulting in the growth of the medical practice; and (3) the rapid and constant changes in the health care environment. Digital technologies are proving to be both a curse and a blessing. On the one hand, they represent yet another source of—often unwanted information [31]—but, on the other hand, they can support physicians in the detection of adverse events and patients at risk of deterioration with the potential to improve health outcomes [32]. This is only achievable if, when planning and implementing digital information solutions in hospitals and clinics, factors such as the provision of clinical training, resources to cope with the additional workload, and optimization of algorithms to reduce superfluous alerts are taken into consideration [32].

In particular, emergency physicians are exposed to further burdens with the need for fast decision making [33], interruptions [34], multitasking [34], and highly variable workloads [35]. Emergency medicine is “a form of medical treatment and aid which seeks to treat unexpected, acute illnesses that pose grave threats to life” [36]. The urgent dimension typical of emergency departments requires doctors to be constantly vigilant, highly reactive, and mentally lucid throughout often very long work shifts [37]. This highly pressurized environment leads to occupational stress, often with negative consequences for doctors’ mental and physical health and work efficiency, which can lead to mistakes in diagnoses and patient treatments [38]. Chronic workplace stress has been identified by the World Health Organization (WHO) as the leading cause of *burnout syndrome* [39]. According to the WHO, the syndrome is characterized by the following 3 main aspects: feelings of exhaustion, feelings of negativism toward one's job, and reduced professional effectiveness [39]. A considerable body of literature has demonstrated that there is a higher prevalence of burnout among emergency physicians compared with other medical professions and specialties [36,40-42]. It is also evident that information overload plays a determinant role in exacerbating burnout in the medical profession [43,44]. However, only 1 study, conducted by Walton [45] on hospital doctors and pharmacists, has considered the impact of information overload beyond burnout on hospital medical staff. The complex setting in which emergency physicians act and operate requires a better understanding of the impact of information overload on their practice and its implications for both patient care, work efficiency, and work/life balance. To the authors’ knowledge, no empirical research has been conducted on information overload as experienced by EMPs specifically and according to different roles, age, and level of seniority; therefore, this paper hopes to serve as a pathbreaker for future studies.

### Aim and Objectives

This study aimed to identify and assess the main causes and sources of medical information overload as experienced by EMPs. Specifically, the research objectives (ROs) were as follows:

1. RO1: Determine the extent to which EMPs experience medical information overload.
2. RO2: Establish the contributory factors to information overload and evaluate their impact on physicians’ working lives.
3. RO3: Identify the coping mechanisms adopted by EMPs to respond to information overload.
4. RO4: Explore whether EMPs at different career stages respond to information overload in different ways.
5. RO5: Determine the impact of physicians’ information overload on patient care.

## Methods

### Data Collection

The assessment of EMPs’ experience with information overload was undertaken using a survey-based primary data collection tool. This was a multicenter study covering 4 NHS trusts in north England, which had approached the staff in their emergency departments and asked them to complete a web-based survey over a period of 3 months in the summer of 2019. All eligible doctors from the trusts were sent an email inviting them to participate in the study. The opening page of the survey included an information sheet and a consent form. Participants were not able to proceed with the survey unless they had ticked the consent option. The study followed the NHS Health Research Authority protocol and was granted both the NHS Research Ethics Committee and the University of Sheffield ethics approvals. The survey, designed using *LimeSurvey GmbH*, took approximately 10 min to complete and was divided into 2 main sections: the first related to demographics and the second addressing emergency physicians’ views on medical information overload, opening with a definition of information overload to guarantee the homogeneity of interpretation. This section was composed of 42 Likert scale–like questions, comprising 2 overarching questions and 4 main aspects of information overload: (1) impact rating of individual information overload sources, (2) impact of information overload on specific work/life aspects, (3) preventative measures to manage information overload, and (4) useful sources of medical information. This part included statements such as “I feel that information overload significantly impacts on my work” and “E-mails I receive are a major contributing factor to my feelings of information overload” rated on a scale from 1 (completely disagree) to 7 (completely agree). There were also 7 open-ended questions to allow the respondents to elaborate on their opinions and perceptions. Most of the statements in the survey were extracted from a literature review conducted by Hall and Walton [8] and from a subsequent survey that one of the authors of this paper (GW) administered to hospital pharmacists and doctors in 2013 [45]. The remaining statements have been informed by 2 other authors (JW and JB) who are directly involved in emergency medicine and are aware of the current main sources of information overload in this sector. In total, the survey invite was sent to 263 physicians; 164 respondents participated with 101 recording full responses (38% response rate), which were then selected for analysis, as 63 records only contained partial demographic information and were deemed unusable.

### Data Analysis

All data were saved in an IBM SPSS version 26 file for analysis. The dataset was initially inspected for errors and out-of-range values for each variable. The demographics were then produced to profile the sample. Next, descriptive statistics were performed for the Likert-style questions in the main section of the survey; means and standard deviations were calculated for each of the statements. A principal component analysis (PCA) was performed on the survey statements related to the sources of information overload. Finally, independent samples *t* tests and one-way between-group analyses of variance (ANOVAs) with Hochberg GT2 post hoc tests were conducted to compare mean scores in relation to respondents’ demographics (ie, the current level of employment, age, and the number of years in emergency medicine). The analyses performed in terms of gender and NHS trust did not show any differences between groups; hence, they are not reported here.

In addition, the open-ended questions and closing remarks were manually coded independently by 2 members of the research team using the 6-step approach to thematic analysis by Braun and Clarke [46].

## Results

### Sample Demographic Profile

This section provides a summary of the demographic characteristics of the respondents. The sample shows a relatively balanced distribution between doctors working as junior trainees (31/101, 30.7%), senior trainees (28/101, 27.7%), and consultants (29/101, 28.7%), whereas 12.9% (13/101) reported other types of working levels (Table 1). In terms of gender, 42.6% (43/101) were male and 56.4% (57/101) were female (only 1/101, 1%, did not indicate their gender). As for age, the majority (70/101, 69.3%) were between 20 and 39 years, with 30.7% (31/101) between 40 and 59 years. In terms of the period since respondents became EMPs, two-third (68/101, 67.4%) had been emergency physicians for 10 years or less. Finally, across the 4 NHS Foundation Trusts surveyed, 39.6% (40/101) of respondents were based at Leeds Teaching Hospitals, followed by Bradford Teaching Hospitals (24/101, 23.7%) and Airedale (23/101, 22.8%), whereas 13.9% (14/101) were based on North Tees and Hartlepool.

**Table 1:** Demographic data from the respondents to the survey.

Demographic characteristics			Values, n (%)
**Age (years)**			
	20-29	24 (23.8)	
	30-39	46 (45.5)	
	40-49	18 (17.8)	
	50-59	13 (12.9)	
**Gender**			
	Male	43 (42.6)	
	Female	57 (56.4)	
	Prefer not to say	1 (1.0)	
**How long have you been an emergency medicine physician for? (years)**			
	<1	21 (20.8)	
	1-4	25 (24.8)	
	5-10	22 (21.8)	
	11-15	16 (15.8)	
	16-20	9 (8.9)	
	>20	8 (7.9)	
**What is your current level?**			
	Junior trainee^a^	31 (30.7)	
	Senior trainee^b^	28 (27.7)	
	Consultant	29 (28.7)	
	Other^c^	13 (12.9)	
**What NHS^d^ trust do you work for? **			
	North Tees and Hartlepool NHS Foundation Trust	14 (13.9)	
	Bradford Teaching Hospitals NHS Foundation Trust	24 (23.7)	
	Leeds Teaching Hospitals NHS Trust	40 (39.6)	
	Airedale NHS Foundation Trust	23 (22.8)	

^a^Foundation year, VTS, and ACCS.

^b^ST4-ST7, staff grade, and junior clinical fellow.

^c^Including advanced clinical practitioner and associate specialist.

^d^NHS: National Health Service.

### RO1 and RO2: Emergency Physicians’ Views on Medical Information Overload

Table 2 summarizes the responses to the Likert scale statements listed in the second section of the survey in terms of mean and standard deviation. The table reports the 2 general questions asked at the beginning of the survey, and the remaining statements were divided across the 4 main information overload aspects covered in decreasing order of importance. The results suggest that there is overall agreement among emergency physicians that information overload has an impact on their work (mean 5.10, SD 1.25), and the problem has been increasing in time (mean 5.63, SD 1.34).

In terms of the sources that have the most impact on medical information overload, there is a wide range of mean values. Doctors listed the 24/7 culture (mean 5.40, SD 1.56), email handling (mean 4.86, SD 1.80), and, in equal measure, multidisciplinary communications (mean 4.51, SD 1.61) and local clinical guidelines (mean 4.50, SD 1.52). On the other hand, the information derived from patients (mean 3.29, SD 1.62), social media (mean 3.15, SD 1.88), and drug companies (mean 2.36, SD 1.50) does not pose particular concerns.

**Table 2:** Emergency clinicians’ perspectives on information overload.

Statements^a^		VMean (SD)
To what extent does medical information overload impact your work?		5.10 (1.25)
To what extent do you agree that medical information overload has increased for you at work?		5.63 (1.34)
**Impact of each of the following causes of medical information overload**		
	24/7 culture (ie, the *always available* workplace)	5.40 (1.56)
	Email	4.86 (1.80)
	Multidisciplinary communication	4.51 (1.61)
	Local clinical guidelines	4.50 (1.52)
	Info from employer/manager	4.43 (1.67)
	Evidence-based practice	4.22 (1.57)
	National clinical guidelines	4.21 (1.52)
	Info to employer/manager (eg, as part of reporting activity)	4.14 (1.67)
	Journal papers	3.41 (1.67)
	Patients bringing information	3.29 (1.62)
	Social media	3.15 (1.88)
	Drug company information	2.36 (1.50)
**How does medical information overload impact on the following aspects?**		
	Guideline fatigue	6.08 (1.56)
	Stress and tension	5.25 (1.37)
	Longer working hours	4.87 (1.70)
	Impaired decision making	4.85 (1.57)
	Imprecise clinical judgments	4.84 (1.45)
	Tiredness/illness	4.81 (1.54)
	Decrease in social life	4.26 (1.80)
**What preventative measures do you adopt to combat information overload?**		
	Prioritization	6.07 (1.13)
	Email handling skills (ie, ability to discriminate by importance)	5.39 (1.50)
	Effective time management	5.30 (1.22)
	Effective information management	5.23 (1.30)
	Software/hardware	3.40 (1.81)
	Information technology point of need delivery	3.13 (1.82)
	Medical librarian	1.85 (1.32)
**What sources do you find most useful when in need of medical information?**		
	Local clinical guidelines	6.13 (1.03)
	National clinical guidelines	5.80 (1.09)
	Colleagues	5.69 (1.34)
	Evidence-based practice	5.14 (1.37)
	Multidisciplinary communication	4.41 (1.49)
	Journal papers	3.54 (1.39)
	Patients bringing information	2.67 (1.56)
	Social media	2.62 (1.65)
	Medical librarian	2.60 (1.60)
	Drug company information	2.17 (1.29)

^a^All statements were rated on a Likert scale from 1 to 7, and the values reported here are in decreasing order of importance.

Views on the consequences of information overload on different aspects of physicians’ daily job and life show a more compacted set of values (less than 2 points difference between the top and bottom entries), indicating that all listed aspects are negatively impacted. Guideline fatigue is the leading consequence of information overload (mean 6.08, SD 1.56), followed by stress and tension (mean 5.25, SD 1.37). However, longer working hours, inaccurate decision making and clinical judgment, and chronic tiredness leading to illness also returned high mean values. Physicians also complained that information overload is detrimental to their personal and social lives (mean 4.26, SD 1.80).

### RO3: Coping Mechanisms Adopted by Emergency Medicine Physicians to Address Information Overload

When it comes to the adoption of preventive measures to overcome information overload, prioritization is by far the most common approach (mean 6.07, SD 1.13), closely followed by email handling (mean 5.39, SD 1.50) and time (mean 5.30, SD 1.22) and information (mean 5.23, SD 1.30) management. Conversely, *external* support such as information technology (IT) and ICT and clinical librarians are not considered particularly effective.

In situations of actual need for medical information, both local (mean 6.13, SD 1.03) and national (mean 5.80, SD 1.09) clinical guidelines are the 2 primary sources, which interestingly highlight the struggle doctors regularly experience. On the one hand, such guidelines are crucial to maintaining a critical level of medical knowledge, but, on the other hand, because of their sheer volume and constant updating, they represent the main cause of medical information overload. In this context, colleagues are also an important source of easily accessible and reliable medical information while on the ward (mean 5.69, SD 1.34). Patient information (mean 2.67, SD 1.56), social media (mean 2.62, SD 1.65), and drug companies (mean 2.17, SD 1.29) are considered irrelevant sources because of their unqualified or biased nature. However, medical librarians have also scored a low mean value (mean 2.60, SD 1.60), perhaps because their services were not considered to be of immediate relevance to emergency physicians’ very fast case turnover.

PCA with direct oblimin rotation with Kaiser normalization was performed on the 12 statements addressing the main causes of information overload. The Kaiser-Meyer-Olkin value was 0.807, exceeding the recommended value of 0.6 [47], and the Bartlett test of sphericity reached statistical significance (*P*<.001), supporting the factorability of the correlation matrix. The analysis identified the presence of 3 factors explaining 42.2%, 12.2%, and 9.6% of the variance, respectively. Table 3 reports the factors and Cronbach α values calculated to assess the reliability of the scale.

**Table 3:** Exploratory factor analysis of factors relative to causes of information overload.

Factors/statements		Cronbach α
**Factor 1: the dutiful physician**		.800
	24/7 *always available* culture	
	Emails	
	Information from employer/manager	
	Information to employer/manager	
	Multidisciplinary communications	
**Factor 2: the informed physician**		.842
	Local clinical guidelines	
	National clinical guidelines	
	Evidence-based practice	
**Factor 3: the current physician**		.785
	Journal papers	
	Social media	

The first factor, named *the dutiful physician*, includes the potential sources of information overload that cannot be ignored or overseen as they form part of physicians’ daily administrative tasks that must be undertaken in addition to direct patient care. These tasks are demanding and time-consuming. Regular reporting to and from line managers or employers and multidisciplinary communications are crucial to the smooth running of emergency departments and provide a more coordinated approach to patient care discharge planning [48]. Most of such communications are exchanged via email, which becomes the main conduit of information overload, and are carried out at all times, hence exacerbating further the *always available* culture.

The second factor, named *the informed physician*, relates to physicians’ need to stay up to date with the changes in guidelines, both at the local and national level and evidence-based practice on how health care professionals should care for patients with specific conditions, and which are based on best evidence practice. These guidelines help the standardization of care across NHS trusts and can be modified to adjust to local needs to provide a cost-effective approach to care [49].

The third factor, named *the current physician*, includes additional sources of information overload that physicians experience on a regular basis in the form of notifications and which are mostly derived from scientific publications (eg, notifications of new articles, subscription invites, and electronic book updates) and social media. The respondents’ comments on this aspect have shown how there appears to be an expectation that physicians, trainees in particular, cluster in online study groups and follow relevant Twitter handles such as #MedEd or #FOMed.

### RO4: Information Overload Perceptions by Age and Years Spent in Emergency Medicine

Independent sample *t* tests have been undertaken on the bank of Likert scale statements in terms of age of respondents and their experience in emergency medicine. All statistically significant results are reported in Tables 4 and 5. In terms of age (Table 4), younger physicians appeared less worried about their reporting activities (mean 3.89, SD 1.02) than older ones (mean 4.65, SD 1.11). Conversely, they are more affected by the loss in social activities because of information overload (mean 4.52, SD 1.31) as opposed to more experienced physicians (mean 3.68, SD 1.05). In addition, younger physicians reported a higher rate of impaired decision making (mean 5.19, SD 1.40) and imprecise clinical judgments (mean 5.12, SD 1.18) than their older colleagues (mean 4.07, SD 1.14 and mean 4.23, SD 1.37), who are still concerned about their performance but have greater experience to draw upon. Younger physicians also seem to rely more on local clinical guidelines (mean 6.28, SD 1.08) than older ones (mean 5.81, SD 1.23) to reduce decision fatigue.

**Table 4:** Independent samples *t* tests on the age of respondents.

Questions	*t* test (*df*)	*P* value	Mean difference	Age (20-39 years), mean (SD)	Age (40-59 years), mean (SD)	Total mean
Impact of info to employer/manager (eg, as part of reporting activity) on medical information overload	−2.109 (94)	.04	0.75	3.89 (1.02)	4.65 (1.11)	4.14
How does medical information overload impact on decrease in social life?	2.098 (51.011)	.04	0.84	4.52 (1.31)	3.68 (1.05)	4.26
How does medical information overload impact on impaired decision making?	3.304 (50.699)	.002	1.12	5.19 (1.40)	4.07 (1.14)	4.85
How does medical information overload impact on imprecise clinical judgments?	2.762 (49.767)	.008	0.89	5.12 (1.18)	4.23 (1.37)	4.84
What preventative measures do you adopt to combat information overload? Email handling skills (ie, ability to discriminate by importance)	−2.055 (70.489)	.04	0.61	5.19 (1.22)	5.81 (1.18)	5.39
What sources do you find most useful when in need of medical information? Local clinical guidelines	2.198 (60.325)	.03	0.47	6.28 (1.08)	5.81 (1.23)	6.13

In terms of years of experience in emergency medicine (Table 5), the only dissimilarity seems to relate to the impact and handling of work emails. Physicians with more than 10 years of experience identify the number of emails received as one of the main causes of information overload (mean 5.39, SD 1.19) as opposed to physicians with less experience who scored a mean of 4.60 (SD 1.26) and hence attributed more importance to email handling skills as a way to manage the problem (mean of 6.03 and SD 1.15 against a mean of 5.06 and SD 1.13 reported for less experienced physicians).

**Table 5:** Independent samples *t* tests on the years spent in emergency medicine.

Questions	*t* test (*df*)	*P* value	Mean difference	Years of experience (0-10 years), mean (SD)	Years of experience (more than 10 years), mean (SD)	Total mean
Impact of emails on information overload	−2.134 (65.084)	.04	0.79	4.60 (1.26)	5.39 (1.19)	4.86
What preventative measures do you adopt to combat information overload? Email handling skills (ie, ability to discriminate by importance)	−3.166 (96)	.002	0.97	5.06 (1.13)	6.03 (1.15)	5.39

### RO4: Information Overload Perceptions by Level of Employment

To conclude the quantitative assessment of the data collected, one-way between-group ANOVAs by the level of employment were also performed on the survey statements (Table 6). Across the 3 main levels populating emergency departments, senior trainees and consultants are those acknowledging the increased information overload with time the most, possibly because of their longer service in hospitals. However, in relation to other aspects, senior trainees seem to experience the most difficulties. For example, in terms of the aspects of their work and life impacted by information overload, they complain about a decrease in social life and diminished decision-making abilities.

**Table 6:** One-way between-group analysis of variance on current level of employment.

Questions	*F* test	*P* value	Junior trainee, mean (SD)	Senior trainee, mean (SD)	Consultant, mean (SD)	Total mean
To what extent do you agree that medical information overload has increased for you at work?	5.798	.004	5.07^a,b^ (1.01)	6.07^a^ (1.02)	6.03^b^ (1.22)	5.63
How does medical information overload impact on decrease in social life?	3.249	.04	4.50 (1.27)	4.79^c^ (1.06)	3.72^c^ (1.36)	4.26
How does medical information overload impact on impaired decision making?	4.331	.02	5.17 (1.14)	5.36^c^ (1.14)	4.29^c^ (1.40)	4.85
What preventative measures do you adopt to combat information overload? Email handling skills (ie, ability to discriminate by importance)	9.223	<.001	5.47 (1.16)	4.67^c^ (1.18)	6.10^c^ (1.01)	5.39
What sources do you find most useful when in need of medical information? Social media	9.246	<.001	2.03^a^ (1.21)	3.59^a,c^ (1.02)	2.23^c^ (1.19)	2.62

^a^Junior trainee versus senior trainee.

^b^Junior trainee versus consultant.

^c^Senior trainee versus consultant.

In addition, whereas for consultants, the ability to effectively manage work emails is crucial to handle, at least partially, information overload (mean 6.10, SD 1.01), senior trainees are the least concerned about this aspect (mean 4.67, SD 1.18). Finally, although the use of social media as a source of medical information is not a very popular choice among emergency physicians in general, senior trainees use them significantly more than the other 2 groups, with a mean of 3.59 (SD 1.02), as opposed to 2.03 (SD 1.21) for junior trainees and 2.23 (SD 1.19) for consultants.

### Qualitative Analysis of Open-Ended Questions

The survey contained several open-ended questions and, although they did not form part of the compulsory sections, still attracted a considerable number of spontaneous and insightful comments.

In terms of the length of time physicians have been experiencing information overload, 39.6% (40/101) claimed that the phenomenon had increased in the last 5/6 years; 10.9% (11/101) confirmed that it started before that, about 10 to 15 years ago; and 14.9% (15/101) did not specify a date but listed aspects such as progressing in their career, noting how the pressure shifted from night shifts only to an all-time problem and overload worsening once the email was firmly established within the NHS. Around 3.0% (3/101) felt that they did not have enough experience to judge the problem, and the remaining respondents did not comment.

The remaining comments revolved around 2 main themes (Table 7), specifically, the ways in which medical information overload impacts physicians’ work and various preventative measures to overcome information overload. The 5 subthemes identified within each of the 2 main themes are discussed below.

**Table 7:** Overview of the themes and subthemes emerging from open-ended questions.

Theme and subthemes		Strands
**Ways in which medical information overload impacts on clinicians’ working life**		
	1. Positive impact	
		Reverse views
	2. Direct causes leading to reduced patient care	
		Impact on patients
		Distraction and forgetfulness
		Confusion
		Delayed decision making
		Tiredness, stress, and anxiety
	3. Indirect causes leading to reduced patient care	
		Lack of time
		Email handling
		Information and communication technology systems
		Breadth of information
		Guidelines and evidence-based practice
		Ability to stay up to date
		Seniority
**Preventative measures to combat information overload**		
	4. Personal attitudes	
		Avoidance
		Therapy
		Work-life balance
	5. Workload management	
		Email and social media management
		Task management
		Time management
		Prioritization
		Delegation

### RO5: Ways in Which Medical Information Overload Impacts Physicians’ Working Life

#### 1. Positive Impact

A total of 3 respondents, all of them consultants, admitted that information overload was indeed present in their working life but did not represent a hindrance to their performance (*reverse views* subtheme):

It doesn’t. You do have be to discerning about where you get information from and use trusted sources though.

Increase in research is interesting and gives more confidence in decision-making. I find this is helpful rather than a hindrance.

The more information I have the better I can do my JOB!

The majority of respondents however did not agree with this view and listed a series of impeding aspects, which can be divided into direct and indirect causes leading to a reduction in patient care.

#### 2. Direct Causes Leading to Reduced Patient Care

Among the direct causes, concerns for the well-being and quality of care of patients (*impact on patients* strand) were the most mentioned and most concerning for the physicians:

On a busy shift on the shop floor when there are competing priorities for my attention, increasingly feel that this means if I try to see my own patients then I either give them poor care or lose control of the department.

Slow decision-making, increased risk of errors.

This continuous feeling of apprehension and an impending sense of loss of control are accompanied by *distraction and forgetfulness*, which was the second most reported issue (strand) caused by information overload and can be summarized in the quote below:

I have a significantly higher background level of anxiety at work due to concerns about giving poor advice or forgetting something important due to the volume of information I am trying to process. I find the non-shop floor information much less of a problem.

Other strands, such as *confusion* with the information received, *delayed decision making* and *tiredness, stress, and anxiety* were mentioned in equal measure as contributing to a potentially reduced patient’s quality of care:

Toward the end of the shift my answers take longer to arrive at, I feel like I am even more risk averse and at times feel like I am can become short with staff asking for help.

During busy environments if documents/protocols contain too much information I may struggle to pick up salient points.

#### 3. Indirect Causes Leading to Reduced Patient Care

Among the indirect causes of information overload, leading to a reduced quality of patient care, *lack of time* to review the information properly was reported as one of the most serious problems:

Huge amount of information coming in and little time to process and implement any actions needed.

Not enough time to document all relevant information consistently.

*Email handling* and problems with *ICT systems* were reported as common, overpowering, and frustrating:

Email mainly - almost overwhelming.

Vast numbers of irrelevant or barely relevant emails containing information which will be forgotten immediately as it concerns rare circumstances or conditions never before experienced (and therefore poorly understood).

Multiple IT systems with different passwords and access. Constant emails - usually not relevant. Feel overwhelmed by the day to day IT so I don’t want to access online resources to broaden my knowledge.

Moreover, 3 additional strands, namely *guidelines and evidence-based medicine*, *ability to stay up-to-date*, and *breadth of information*, are related to the need to be informed that physicians experience on a daily basis [50] and are difficult to address in an environment overloaded with what seems like irrelevant information:

I feel I am unable to keep up to date with the multitude of clinical guidelines from NICE, the college...only exacerbate by looking at Tweeter!

The number of guidelines/policies that now exist both locally and nationally - it is hard to keep track of all of these.

Finally, *seniority* was also mentioned as a hindering factor contributing to information overload, as more junior staff and nurses (and patients) tend to direct their queries and doubts to the more experienced physicians:

More difficult out of hours/weekends when I am the only senior decision-maker in the department. If working with a good registrar this can lessen the impact but if working with a less experienced middle grade all questions/information comes though me.

Questions from juniors and other colleagues.

Lots of questions asked throughout shift from nurses to other doctors.

### Preventative Measures to Combat Information Overload

When it comes to addressing and solving the problem of information overload in effective ways, respondents suggested solutions that can be classified into 2 subthemes: *personal attitudes* and *workload management strategies*.

#### 4. Personal Attitudes

As part of the individual attitudes observed, some physicians simply ignored the problem (*avoidance* strand). Some other physicians adopted a very pragmatic approach and had no difficulties recognizing their own limits:

When I am at work and feeling overloaded, I step away from the situation.

Learn to ignore.

Acceptance that you can't do everything.

Psychological stress is increasingly recognized within the medical profession, particularly in emergency medicine [51], and some respondents prefer to rely on formal support in the form of *therapy*:

Therapy - I have had CBT sessions and NLP sessions to help me deal with the stress and anxiety information overload gives me and to gain strategies regarding how to cope with this.

I take little steps and with the help of my therapist I try not to compare myself negatively to others.

Others preferred to self-manage and find a more balanced relationship between working and personal life (*work-life balance* strand), although often long shifts and complex working patterns make this option fairly difficult to attain:

My days off are for fun and time away - I do not work on days off. I avoid seeing colleagues on these days who talk about work when they are not there.

I feel a lot of the ‘critical information’ I need for my day to day job is limited - especially while trying to revise for exams, the need to switch off and rest is very important.

#### 5. Workload Management

Physicians have also indicated a number of strategies to manage information overload that are more strictly linked to practical workload management and dealing effectively with emails and social media (*email and social media management* strand). This appeared to be a priority for many respondents:

When I am not at work or feeling overloaded I ignore my e-mails and mute my social media accounts.

Nil work emails on phone mute work WhatsApp groups when on leave no longer active on Facebook at all due to general overload but also work based groups.

This was followed by strategies to address *task and time management* in an effective and sustainable way:

Keeping a to-do- list booklet.

Summarising things. Writing things down properly helps consolidate my head.

Finally, *prioritization* and *delegation* were also mentioned as effective ways of managing information overload, particularly at times of crisis:

Prioritisation and having some “IT free” time is vital.

Delegation of clinical supervision.

## Discussion

### RO1: Determine the Extent to Which Emergency Medicine Physicians Experience Medical Information Overload

Both quantitative and qualitative data have shown that emergency physicians are deeply affected by an ever-increasing amount of medical information impacting every aspect of their job. The 24/7 (or *always available*) culture places itself firmly at the heart of the problem, as identified by Walton [45], and leads to problems with fatigue and, consequently, with performance, productivity, and safe decision making [52]. All these aspects have been highlighted by physicians in this study as being compromised not just by the very fast-moving pace typical of their job, but by the constant and relentless information received and the information requested from them. What emerged very clearly from this study is the difficulty in striking a balance between the need to maintain an acceptable and current level of knowledge and the ability to sift and discriminate through large amounts of irrelevant information. There are valid arguments supporting both the benefits of information overload and its drawbacks. A small outlier group of respondents composed of 3 consultants claimed how there was no such thing as information overload as a negative attribute of their job. They see information as an opportunity to learn, as observed by Falschlunger [21], and increase their confidence in their decisions, in line with the *freedom of choice* described by Brehm and Brehm [53] and De Angelis [54]. However, the majority of physicians perceived information overload as a negative concept hindering their ability to process information effectively and accurately [55]. The migration toward *big data* handling by health care professionals because of the information expansion phenomenon described by Yamamoto [30] is also reflected in this study as physicians are faced with multiple evolving environments. The increasing number of emails and social media notifications received daily and regular updates in policies and evidence-based practice, coupled with new or updated IT systems to keep abreast of, will all contribute to feelings of inadequacy and frustration. Unless controlled and harnessed, such feelings, in the long term, can lead to *burnout syndrome*, which is very common among all types of health care professionals [43,44]. It is difficult to establish when physicians have started experiencing the burden of information overload in their careers. It appears that consultants and people working in emergency departments the longest have noticed a steady increase, made sharper since the ready access to internet-based resources and emails started over 20 years ago. This is in line with findings by Roetzel [56], who reported how the research around the 1980s and, particularly, the 1990s coincided with the rise and spread of information technologies. However, for most, the problem started to take shape with the advent of mobile platforms and the deployment of electronic health record systems in NHS trusts in 2005 [57].

### RO2: Establish the Contributory Factors to Information Overload and Evaluate Their Impact on Physicians’ Working Life

The leading causes of information overload in emergency physicians are multiple, and it is often difficult to discriminate among them because of their interlinked nature. The PCA conducted on the survey statements revealed 3 main factors contributing to information overload. The main factor leading to the concept of *the dutiful physician* is the administrative tasks physicians must carry out as part of their daily routine, such as reporting, responding to emails, and communicating with the other physicians forming a patient’s care team. This study has reported an increased burden because of email handling compared with Walton’s [45] research on hospital doctors, leading to the conclusion that, in the last 6 years, since that study was published, the number of emails has significantly increased as part of physicians’ daily duties. It is also clear that administrative tasks are one of the main causes of physicians’ burnout syndrome [58] because of their persistent and repetitive nature. However, physicians are also aware of the importance of carrying out such tasks for the smooth running of the ward and the good care of patients [26]. A second set of causes deeply affecting physicians’ perceptions of information overload is linked to the consultation of local and national clinical guidelines. Ironically, such guidelines, as important as they are to guarantee patients’ consistency of care, are also a burden to health care professionals because of their frequent updates and notifications. On the one hand, physicians must remain current (ie, *the informed physician*) and follow the policies and directives reported in the guidelines to guarantee the quality of patient care, but on the other hand, the pressure of being up to date and making sure nothing is overlooked plays a considerable role in increasing physicians’ stress [59]. A third factor contributing to information overload entails the number of notifications and alerts received mostly via social media, but also from journal editors and publishers and regarding new medical approaches, clinical trials, discoveries, and case studies (*the current physician*). These sources have registered a significant increase since Walton’s study [45] in 2014, likely because of the current much wider diffusion of social media platforms in all aspects of society. Once again, physicians experience an internal dispute about when to start ignoring such alerts. Maggio and Artino [59] believe that medical librarians could help battle physicians’ information overload and help them navigate through the various systems and social media, but they are not utilized as much as they should, a conclusion corroborated by this study, where librarians score among the least useful sources of medical information (Table 2). However, regardless of the causes of information overload, the consequences can be summarized as directly or indirectly leading to reduced patient care, which will be discussed further below.

### RO3: Identify the Coping Mechanisms Adopted by Emergency Medicine Physicians to Respond to Information Overload

To help mitigate information overload, physicians have devised strategies for managing and filtering available medical information. The results of this study have shown similar results and led to the conclusion that information overload prevention and mitigation are left entirely in the hands of individuals and their personal views and preferences. A number of actions are taken from a pragmatic point of view (workload management) quite regularly, but with varying degrees of success. Among the most common approaches, email and social media handling skills are highly desirable, considering the steadily growing amount of communication, including patient information, passing through these tools [60]. Strictly linked to this is also the ability to prioritize tasks (including emails and social media), appearing very high in the list of possible solutions, particularly in situations of multiple concurrent demands [33], which are common practices in emergency departments. Prioritization is a skill developed with time and experience [61], in situations of high demand and variable workload [34], and is particularly popular in emergency departments. Task, time, and information management are other measures frequently adopted by physicians to overcome medical information overload and can take the form of simple actions, such as keeping daily and weekly to-do lists, maintaining written records, timing actions, summarizing information, or key articles to help consolidate knowledge. Although most of the above points were identified by Walton [45], in this study, their importance is stressed further, and new approaches have emerged, including those dictated by personal attitudes. These play an important role in determining how people deal with information overload. This study has highlighted a few approaches, although not all adopted with the same conviction. A small group of respondents admitted that the easiest way is to ignore circumstances or demands when feeling overloaded, which is not necessarily a positive action to undertake, particularly in emergency care. This is symptomatic of the difficulty and pressure physicians are subject to and is a common self-preservation mechanism triggered by overwhelming situations [62]. Other people have mentioned formal therapy and a better work-life balance in which working and private life are kept separate, and personal time is seen as an opportunity to regain strength and stability. These approaches are not new, nor specific to emergency physicians, as they are common to most workplaces [11,19], but it would be advisable to integrate them with formal training on the job and regular sessions on developing coping skills.

### RO4: Explore Whether Emergency Medicine Physicians at Different Career Stages Respond to Information Overload in Different Ways

This part of the study offers a new viewpoint of medical information overload drawn from physicians’ experiences at different times in their careers and highlights deeply diverse perceptions and needs.

The study revealed an overall homogeneity of perceptions of medical information overload, in the sense that most respondents agreed on its increasing presence, size, and impact. However, some specific aspects showed differences in responses depending on the seniority of the physician. For example, people who have been in emergency care for 10 years or longer appeared to resent the increased number of emails received and attributed more importance to the ability to handle them than people who have been working in emergency care for less than ten years. This is also in line with the independent sample *t* tests conducted on the age of the respondents and with the ANOVA tests conducted on the level of employment. However, younger physicians and trainees appeared to be more worried about the loss in social life, impaired decision making, and imprecise clinical judgment than older colleagues and consultants. The literature demonstrates that junior clinicians have higher levels of anxiety because of uncertainty than senior clinicians [63,64], and this is reproduced in this study because of the additional stress of information overload. In particular, it appears that senior trainees are the most concerned about this problem and are the most affected by it, particularly with respect to impaired decision making. A recent study on Canadian emergency medicine residents [65] revealed how junior trainees are properly supervised and supported, but when they then become senior trainees, they lose most of that support and are left to fend for themselves. In addition, senior trainees are at a very demanding stage in their career, where they are preparing for independent practice [66] and are entrusted with more complex decisions than ever before. This is bound to contribute to an increased sense of unease and frustration when it comes to information overload.

### RO5: Determining the Impact of Physicians’ Information Overload on Patient Care

Compromising the quality of patient care represents the ultimate consequence of medical information overload and rests at the forefront of physicians’ thoughts. The qualitative responses in this study reported on the physicians’ perceptions of the problem and showed how slow decision making because of information overload and frequent interruptions can lead to substandard care and errors in diagnoses and prescriptions. In addition, remembering patient diagnostics and medical history becomes difficult throughout a shift because of the large amount of information acquired about other cases and other confounding factors [28]. The fast turnover of emergency departments implies that there can often be a queue of people waiting to speak to a physician, who frequently does not manage to action one of these requests before others come in, hence compromising patient safety [32]. Among the effects of information overload mentioned by the respondents, distraction and tiredness play a key role. Physicians experience a higher background level of anxiety at work because of concerns about giving poor advice or forgetting something important because of the volume of information they are trying to process. This is exacerbated by long shifts, which can leave physicians both mentally and physically drained [43,44]. In critical situations, finding the right information fast is essential, but information overload can prevent this. Some respondents have been struggling with the correct information they should follow, as sometimes a lot of vague or unclear guidelines or advice can be worse than none at all, whereas others complained that documents and protocols contained so much information that it was difficult to discern salient points. In addition to this, other less obvious causes can still lead to poor patient care, particularly the ubiquitous nature of digital technologies, which provoke conflicting feelings among health care professionals. They are perceived as intrusive and time-consuming, but also as an important resource of clinical information, particularly when dealing with emergencies [31]. A recent study by Laker et al [28] introduced the concept of *emphasis framing* to aid health care professionals toward a more focused approach to decision making. This cognitive process could help in identifying the most relevant components of any piece of information while discarding the rest. For example, physicians could be directed toward article abstracts rather than full papers or be alerted to an incorrectly entered value for a medication prescription. The study reported an increase in clinical decision-making time through this approach, but also a significantly improved quality of medical decisions. Although a lot of work needs to be done to properly assess the real benefits of emphasis framing and how to implement it in real-world workflows, it is a research direction worth exploring further.

### Conclusions

This study reported on the high and ever-increasing medical information overload in emergency medicine departments in the UK NHS trusts. Among the many factors listed by physicians as influencing information overload, the historical emergency medicine culture of being constantly available throughout a shift (24/7 culture), email, and multidisciplinary communications have been mentioned as being the most severe. However, different levels of employment, experience in emergency medicine, and age also seem to influence physicians’ perceptions of the leading causes of information overload. Nevertheless, some information is critical to guarantee high standards of patient care, particularly those derived from clinical guidelines.

This study advances previous research on information overload in 4 distinct ways: (1) by identifying the current causes affecting UK emergency medicine while also recognizing key information sources; (2) by offering insights on how information overload affects physicians in relation to demographic factors such as age, years spent in emergency medicine, and level of employment (ie, seniority); (3) by identifying mechanisms that emergency physicians put in place to manage information overload; and finally (4), by categorizing influencing factors in terms of their impact on patient care and reports on preventative measures currently in use to combat information overload.

However, this study is also subject to the following limitations: (1) although survey is an effective method to receive responses from a large number of participants and to explore relationships between variables, it might be less effective to answer complex questions such as user perceptions of information overload, contributing factors, and coping mechanisms, particularly when limited previous studies were conducted on this topic and with this particular user group; (2) the sample size is limited, although studies conducted on health care professionals are usually affected by this problem; (3) some of the variables listed as potential sources of information overload can be difficult to uniquely discriminate from each other (eg, emails may include clinical guidelines, employer information, and patient information), but the survey wanted to offer both information content aspects and information source channels; and (4) the study was only able to assess physicians’ *perceptions* on the impact that information overload has on the quality of patient care. To attain an objective measure of such impact, different indicators would have been needed to allow for a direct correlation between patients’ health outcomes and physicians’ information overload.

This study has identified further research areas that could be worth taking forward to provide a more in-depth and more informed insight into information overload. The 24/7 culture, multidisciplinary interactions, and the pressure of following relevant guidelines are aspects specific to the emergency medicine profession and must be addressed to relieve physicians. Further investigation could determine the influence of professional boundaries on such aspects. A consistent thread in the findings was the contribution made by email in increasing information overload. This was an interesting outcome where it could be assumed that social media as a communication channel was taking the place of email. This would point to the value of a more detailed exploration, specifically on email and information overload. An area of concern that this study established was the interrelationship between clinical guidelines and information overload. By establishing how clinical guidelines make this contribution, it is possible to determine how presentation, style, and format could be adapted to make them more accessible.

NHS trusts in the United Kingdom should consider some of the strategies adopted by the physicians participating in this study to reduce the burden of information overload (eg, prioritization) and try to embed routine training or guidance on time and information management skills in staff development programs.

## Abbreviations

ANOVA: analysis of variance
EMP: emergency medicine physician
ICT: Information and Communication Technology
IT: information technology
NHS: National Health Service
PCA: principal component analysis
WHO: World Health Organization
